# Clathrin- and Dynamin-Independent Endocytosis of FGFR3 – Implications for Signalling

**DOI:** 10.1371/journal.pone.0021708

**Published:** 2011-07-14

**Authors:** Ellen Margrethe Haugsten, Malgorzata Zakrzewska, Andreas Brech, Sascha Pust, Sjur Olsnes, Kirsten Sandvig, Jørgen Wesche

**Affiliations:** 1 Centre for Cancer Biomedicine, Faculty Division Norwegian Radium Hospital, University of Oslo, Oslo, Norway; 2 Department of Biochemistry, Institute for Cancer Research, The Norwegian Radium Hospital, Oslo, Norway; 3 Faculty of Biotechnology, University of Wroclaw, Wroclaw, Poland; Thomas Jefferson University, United States of America

## Abstract

Endocytosis of tyrosine kinase receptors can influence both the duration and the specificity of the signal emitted. We have investigated the mechanisms of internalization of fibroblast growth factor receptor 3 (FGFR3) and compared it to that of FGFR1 which is internalized predominantly through clathrin-mediated endocytosis. Interestingly, we observed that FGFR3 was internalized at a slower rate than FGFR1 indicating that it may use a different endocytic mechanism than FGFR1. Indeed, after depletion of cells for clathrin, internalization of FGFR3 was only partly inhibited while endocytosis of FGFR1 was almost completely abolished. Similarly, expression of dominant negative mutants of dynamin resulted in partial inhibition of the endocytosis of FGFR3 whereas internalization of FGFR1 was blocked. Interfering with proposed regulators of clathrin-independent endocytosis such as Arf6, flotillin 1 and 2 and Cdc42 did not affect the endocytosis of FGFR1 or FGFR3. Furthermore, depletion of clathrin decreased the degradation of FGFR1 resulting in sustained signalling. In the case of FGFR3, both the degradation and the signalling were only slightly affected by clathrin depletion. The data indicate that clathrin-mediated endocytosis is required for efficient internalization and downregulation of FGFR1 while FGFR3, however, is internalized by both clathrin-dependent and clathrin-independent mechanisms.

## Introduction

Signalling from receptors at the cell surface is regulated by endocytosis. For instance, signalling from many receptors is terminated by internalization and degradation in lysosomes. Furthermore, an endosomal location can allow the receptors to recruit and activate downstream signalling molecules different from the cell surface receptors [1]–[3]. Thus, internalization is an important step which can influence the duration of signalling as well as the specificity of signalling targets.

Several pathways of internalization that differ in the required protein machinery have been described. The best studied endocytic mechanism is characterized by the formation of clathrin coated pits at the plasma membrane. The pits pinch off from the cell surface by the large GTPase dynamin. Internalization of many receptors such as the transferrin (Tf) receptor and the epidermal growth factor receptor (EGFR) are mainly clathrin dependent [4]–[6]. However, clathrin-mediated internalization of EGFR seems to vary with the conditions. Under conditions of moderate expression, the EGFR are predominantly internalized through clathrin-mediated endocytosis. Under conditions of overexpression of EGFR or high ligand concentrations, the receptor is internalized through both clathrin-mediated and non-clathrin endocytosis [7].

The clathrin-independent mechanisms of internalization are less well understood than the clathrin-dependent mechanism. A classification of the different clathrin-independent mechanisms into five potential subgroups has been proposed: caveolar, IL2-receptor, GEEC/CLIC, Arf6, and flotillin endocytic pathway [8], [9]. Two of them, the IL2-receptor and the caveolar uptake require dynamin [8]. Several other small GTPases have also been associated with the different clathrin-independent pathways, such as Cdc42 with the GEEC/CLIC pathway. However, the number and molecular identity of clathrin-independent endocytic mechanisms are still not clear and the identification of more specific markers and cargo molecules are needed to allow a definitive dissection of the clathrin-independent endocytic pathways [10]–[12].

It has been suggested that the route of internalization can determine whether signalling receptors are degraded or recycled, and whether or not they initiate signalling from endosomes. It has been proposed that EGFRs internalized via a clathrin-dependent pathway are recycled back to the cell surface whereas EGFRs internalized independently of clathrin are efficiently degraded [13]. Similarly, TGF-β receptors internalized via caveolae are sorted to degradation, whereas those internalized via clathrin-coated pits are directed to an endosomal compartment associated with accessory proteins which promote signal transduction [14], [15]. In this way, the many endocytic pathways are not solely different mechanisms for internalization but can also dictate the further signalling and intracellular trafficking of their cargo.

The fibroblast growth factor receptor (FGFR) family consists of four tyrosine kinase receptors designated FGFR1-4. Upon ligand binding the receptors are autophosphorylated and activate several signalling pathways such as the Ras/MAPK (mitogen-activated protein kinase), phosphoinositide 3-kinase/Akt and PLC-γ (phospholipase C-γ)/protein kinase C [16]. Depending on the target cell type, FGFR signalling can induce cell proliferation, differentiation, apoptosis and cell motility. Deregulation of FGFR signalling has been associated with a number of serious disorders such as cancer and several forms of dwarfism [17]–[19].

The signalling mechanisms of FGFRs have been extensively studied but the internalization pathways, however, are not entirely clear. We and others have found that endocytosis of FGFR1 depends on clathrin [20], [21]. Moreover, a splice variant of FGFR2 called FGFR2b or keratinocyte growth factor receptor (KGFR) was demonstrated to localize to clathrin coated pits and later on, it was reported that depletion of clathrin inhibited KGFR endocytosis [22], [23]. A few studies describe endocytic trafficking and sorting of FGFR3 but the machinery responsible for FGFR3 internalization remains to be elucidated [24], [25]. Since deregulated FGFR3 signalling is involved in a variety of skeletal disorders as well as in certain malignancies, it is important to elucidate how FGFR3 signalling is regulated. Here we characterize the molecular machinery involved in endocytosis of FGFR3 and further examine how internalization influences FGFR signalling.

## Results

### Internalization of FGF1 by FGFR1 and FGFR3 in cells depleted of clathrin heavy chain

In a recent paper we reported that FGF1 is internalized predominantly via clathrin-mediated endocytosis in U2OS cells stably transfected with FGFR1 [20]. In order to characterize the internalization of FGFR3, we generated U2OS cells stably expressing FGFR3. This made it possible to compare the internalization of FGFR3 to that of FGFR1 in the same cellular system and under approximately similar conditions. FGF1 is assumed to bind similarly well to FGFR1 and FGFR3 [26].

**Figure 1:**
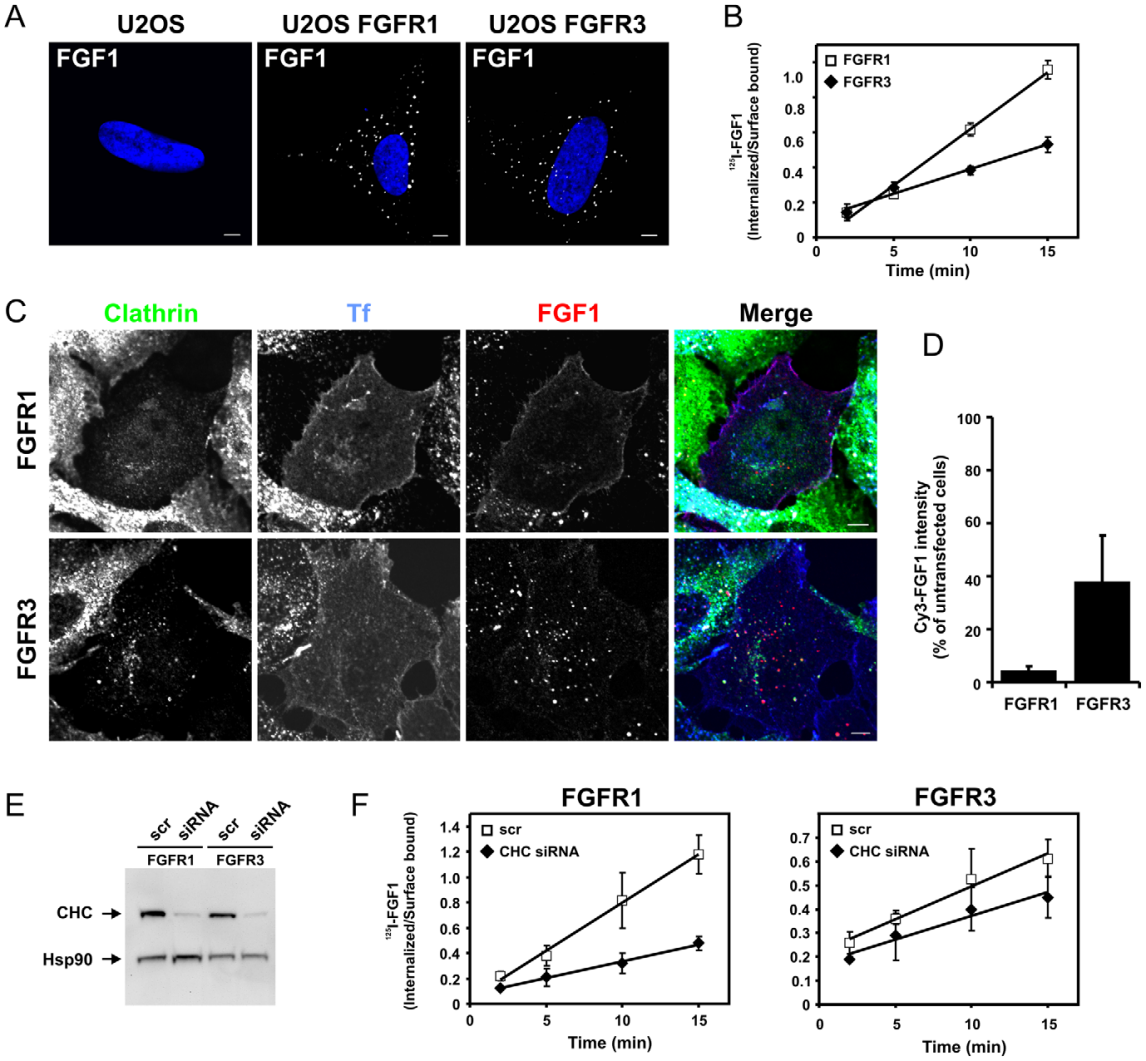
The effect of clathrin heavy chain depletion on endocytosis of FGF1 in cells expressing FGFR1 or FGFR3. (**A**) Untransfected U2OS cells or U2OS cells stably expressing FGFR1 or FGFR3 were incubated for 20 minutes at 37°C with Cy3-FGF1 and 50 U/ml heparin. The cells were stained with Hoechst 33342 and examined with confocal microscopy. Bar, 5 µm. (**B**) U2OS cells stably expressing FGFR1 or FGFR3 were grown on gelatinized plates and incubated with 10 ng/ml ^125^I-FGF1, 20 U/ml heparin and 0.2% gelatine at 37°C for indicated periods of time. Internalized and surface-bound ^125^I-FGF1 were separated as described in *Materials and methods* and the ratio was plotted as function of time. The graph represents the mean ±s.d. of three independent experiments with three parallels. (**B**) U2OS cells stably expressing FGFR1 or FGFR3 were subjected to vector-based siRNA against CHC for 4 days to knock down clathrin. The cells were then incubated for 20 minutes at 37°C with Cy3-FGF1, Alexa 647-Tf and 50 U/ml heparin. The cells were stained with goat anti-CHC antibody and examined with confocal microscopy. Bar, 5 µm. (**C**) The uptake of Cy3-FGF1 was measured as Cy3 intensity in non-depleted and CHC depleted cells treated as described in B. Only cells in which Tf uptake was inhibited were defined as depleted of CHC. The mean intensity of Cy3 in CHC depleted cells is presented in the histogram as the percentage of Cy3 intensity in non-depleted cells. The histogram represents the mean +s.d. of three independent experiments and 15-45 cells were quantified in each case in each experiment. Confocal scanning was performed with identical settings. (**D**) U2OS cells stably expressing FGFR1 or FGFR3 were transfected with siRNA oligos (100 nM) targeting CHC or a non-targeting siRNA control (scr) as described in *Materials and Methods*. The level of knockdown was assessed in every experiment by Western blotting using mouse anti-CHC antibody. Anti-Hsp90 antibody was used as a loading control. One representative Western blot is shown. (**E**) U2OS cells stably expressing FGFR1 or FGFR3 were transfected with siRNA oligos (100 nM) targeting CHC or a non-targeting siRNA control (scr), grown on gelatinized plates and incubated with 10 ng/ml ^125^I-FGF1, 20 U/ml heparin and 0.2% gelatine at 37°C for indicated periods of time. Internalized and surface-bound ^125^I-FGF1 were separated as described in *Materials and Methods* and the ratio was plotted as function of time. Note the different scale on the Y axis. The graph represents the mean ±s.d. of three independent experiments with three parallels.

To exclude the possibility that U2OS cells express endogenous FGFRs that could interfere with the experimental setup, untransfected U2OS cells or U2OS cells stably expressing FGFR1 or FGFR3 were allowed to internalize Cy3-FGF1 for 20 minutes before fixation and examination in a confocal microscope. Cy3-FGF1 was not detected in untransfected U2OS cells as opposed to U2OS cells stably expressing FGFR1 or FGFR3 (Fig. 1A). Moreover, we were not able to detect any of the four FGFRs in untransfected U2OS cells by Western blotting (Fig. S1). These data indicate that the U2OS cells do not express detectable levels of any of the four FGFRs endogenously.

First, we compared the endocytic uptake of ^125^I-FGF1 in cells stably expressing FGFR1 or FGFR3. We noticed that the uptake of FGFR3 was significantly slower than that of FGFR1 (Fig. 1B). The rate of FGF1 internalization was measured as the ratio of endocytosed to surface bound ^125^I-FGF1 at different time points and the slope in Fig 1B for FGFR1 cells is 0.0648 and 0.0283 for FGFR3 cells. The different kinetics for FGF1 uptake via FGFR3 compared to FGFR1 indicates that different endocytic mechanisms might be involved.

Thus, to test whether endocytosis of FGF1 in FGFR3 cells is mediated by clathrin, U2OS cells stably expressing FGFR3 or FGFR1 (as a control) were transfected with vector-based siRNA against clathrin heavy chain (CHC). The cells were then allowed to internalize Cy3-FGF1 and Alexa 647-Tf for 20 min and the cells were fixed and stained with anti-CHC antibody. Subsequently, the cells were examined in a confocal microscope. Cells with efficient knockdown of clathrin were identified based on their low level of anti-CHC antibody staining and their inability to internalize Alexa 647-Tf. In FGFR1 cells depleted of clathrin, little Cy3-FGF1 could be detected inside the cells and a Cy3-FGF1 pattern which resembles cell surface staining was observed (Fig. 1C). This probably represents FGF1 bound to receptors at the cell surface indicating that internalization of FGFR1 is indeed dependent on clathrin. Interestingly, in FGFR3 cells depleted of clathrin, Cy3-FGF1 was clearly still internalized. Also in these cells, the uptake of Alexa 647-Tf was blocked indicating an efficient knockdown of CHC.

The intensity of Cy3-FGF1 staining in cells depleted of clathrin was quantified and compared to the intensity of Cy3-FGF1 staining in cells with normal clathrin levels. In FGFR1 cells depleted of clathrin, the uptake of FGF1 was reduced to about 4% compared to non-depleted cells whereas the uptake of FGF1 in FGFR3 cells depleted of clathrin was only reduced to approximately 40% compared to non-depleted cells (Fig. 1D). These results confirm that FGFR1 is internalized predominantly via clathrin-mediated endocytosis and they demonstrate that endocytosis of FGFR3 is only partly dependent on clathrin.

Similar results were also obtained using another siRNA approach. Cells were depleted of clathrin using a specific oligo-based siRNA against CHC [6] and then the rate of FGF1 internalization was examined in cells expressing FGFR1 or FGFR3 and measured as the ratio of endocytosed to surface bound ^125^I-FGF1 at different time points. Efficient knockdown of clathrin was confirmed by Western blotting (Fig. 1E). The rate of endocytosis of FGF1 in FGFR1 cells depleted of clathrin was significantly reduced compared to cells with intact clathrin levels (Fig. 1F). The slope in Figure 1F for FGFR1 cells was reduced from 0.0761 in FGFR1 cells transfected with scrambled siRNA to 0.0267 in FGFR1 cells depleted of clathrin. This is in agreement with our previous findings [20]. The rate of endocytosis of FGF1 in FGFR3 cells depleted of clathrin was only partly reduced compared to cells with intact clathrin levels. The slope in Figure 1F was reduced from 0.0275 in scrambled siRNA transfected FGFR3 cells to 0.0197 in FGFR3 cells depleted of clathrin. The experiment was repeated in three different clones of U2OS cells stably expressing FGFR3 with similar results (data not shown). Moreover, CHC knockdown did not interfere with the number of receptors at the cell surface at steady state measured as ^125^I-FGF1 bound to the cells (Fig. S2A). Also, the uptake of ^125^I-Tf was reduced to a similar extent in U2OS cells stably expressing FGFR1 or FGFR3 upon clathrin depletion (Fig. S2B).

**Figure 2:**
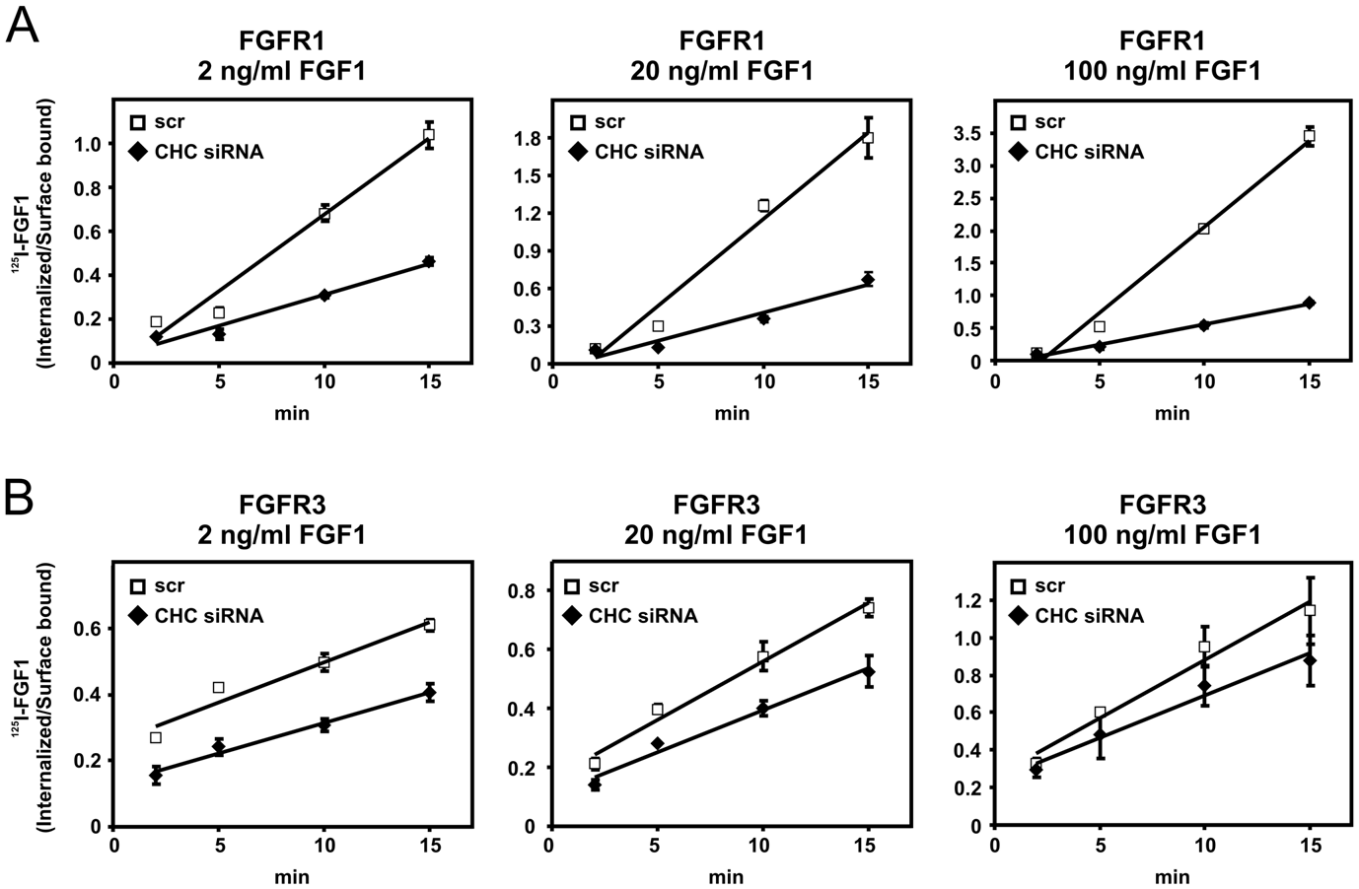
The effect of low and high concentrations of FGF1 on endocytosis in cells expressing FGFR1 or FGFR3 and depleted of clathrin heavy chain. U2OS cells stably expressing FGFR1 (**A**) or FGFR3 (**B**) were transfected with siRNA oligos (100 nM) targeting CHC or a non-targeting siRNA control (scr), grown on gelatinized plates and incubated with 2, 20 or 100 ng/ml ^125^I-FGF1, 20 U/ml heparin and 0.2% gelatine at 37°C for indicated times. Internalized and surface-bound ^125^I-FGF1 were separated as described in *Materials and Methods* and the ratio was plotted as function of time. Note the different scale on the Y axis. The graph represents one independent experiment with three parallels ±s.d.

Sigismund *et al*. [27] reported that EGFR is internalized through clathrin-mediated endocytosis and non-clathrin endocytosis dependent on ligand dose. We therefore investigated if clathrin-mediated endocytosis of FGFR1 and FGFR3 could also be dependent on ligand dose. Saturation binding experiments demonstrated that concentrations of ^125^I-FGF1 above 20–30 ng/ml were saturating in both cell lines (Fig. S3). Therefore, we used 2 ng/ml, 20 ng/ml and 100 ng/ml ^125^I-FGF1 to test if high (100 ng/ml) and low (2 ng/ml) concentrations of FGF1 affect the clathrin-mediated endocytic uptake via FGFR1 and FGFR3. The cells were depleted of clathrin using siRNA oligos and the ratio of internalized to surface bound ^125^I-FGF1 at different time points was examined. As can be seen in Fig. 2, the knockdown of CHC reduced the rate of endocytosis of FGF1 to the same extent whether high or low concentrations of ^125^I-FGF1 were used. This is in agreement with the data presented in Fig. 1 since we used low concentrations of ^125^I-FGF1 to investigate the rate of endocytosis in clathrin knockdown cells whereas high concentrations of Cy3-FGF1 was used in the confocal experiments. In both cases, endocytosis of FGFR1 was dependent on clathrin and endocytosis of FGFR3 was only partly dependent on clathrin.

### Role of dynamin in internalization of FGF1 by FGFR1 and FGFR3

**Figure 3:**
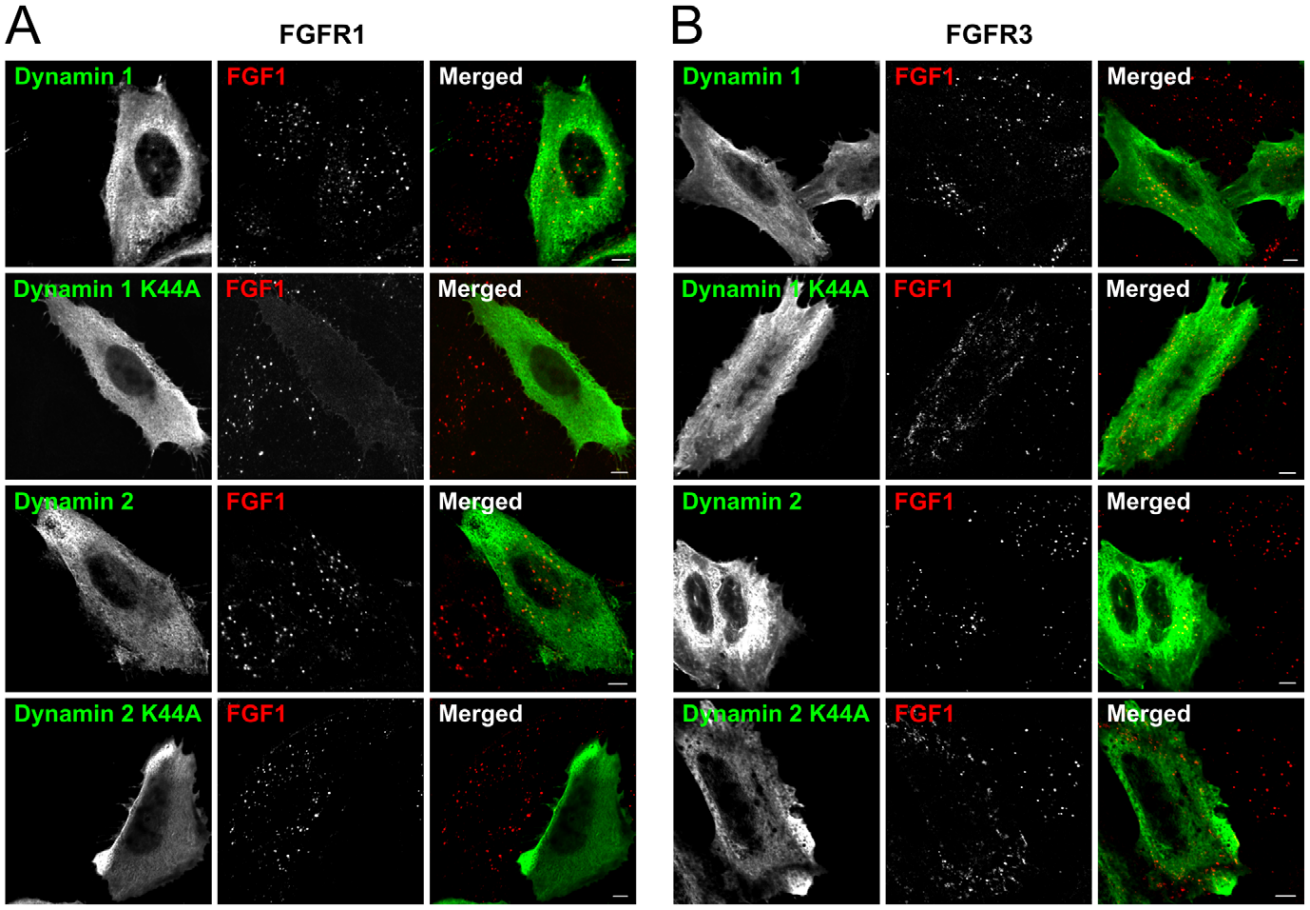
The effect of dynamin 1, dynamin 1 K44A, dynamin 2 or dynamin 2 K44A expression on FGF1 internalization. U2OS cells stably transfected with FGFR1 (**A**) or FGFR3 (**B**) were transfected with HA-tagged dynamin constructs as indicated and incubated with Cy3-FGF1 and 50 U/ml heparin at 37°C for 20 min. The cells were then fixed and stained with anti-HA antibody. The cells were examined with confocal microscopy. Bar, 5 µm.

Many of the endocytic pathways described to date, including clathrin mediated endocytosis, are dependent on the small GTPase dynamin. To test if endocytosis of FGF1 via FGFR3 is dependent on dynamin, U2OS cells stably expressing FGFR3 or FGFR1 (as a control) were transfected with either wild-type dynamin 1 or 2 or a dominant negative mutant of dynamin 1 or 2 (dynamin 1 K44A or dynamin 2 K44A) [28], [29]. The transfected cells were allowed to endocytose Cy3-FGF1 for 20 minutes and then the cells were fixed and analysed by confocal microscopy. The data in Fig. 3A and B demonstrate the presence of intracellular vesicles containing endocytosed Cy3-FGF1 in FGFR1 and in FGFR3 cells expressing wild-type dynamin 1 or 2. In cells expressing the dominant negative constructs of dynamin, the pattern of Cy3-FGF1 was changed. In transfected FGFR1 cells, Cy3-FGF1 was now hardly detectable inside the cells indicating that internalization of FGFR1 is dependent on dynamin. In transfected FGFR3 cells, Cy3-FGF1 was distributed in dots. A similar pattern was observed in three different clones of U2OS cells stably expressing FGFR3 (data not shown).

**Figure 4:**
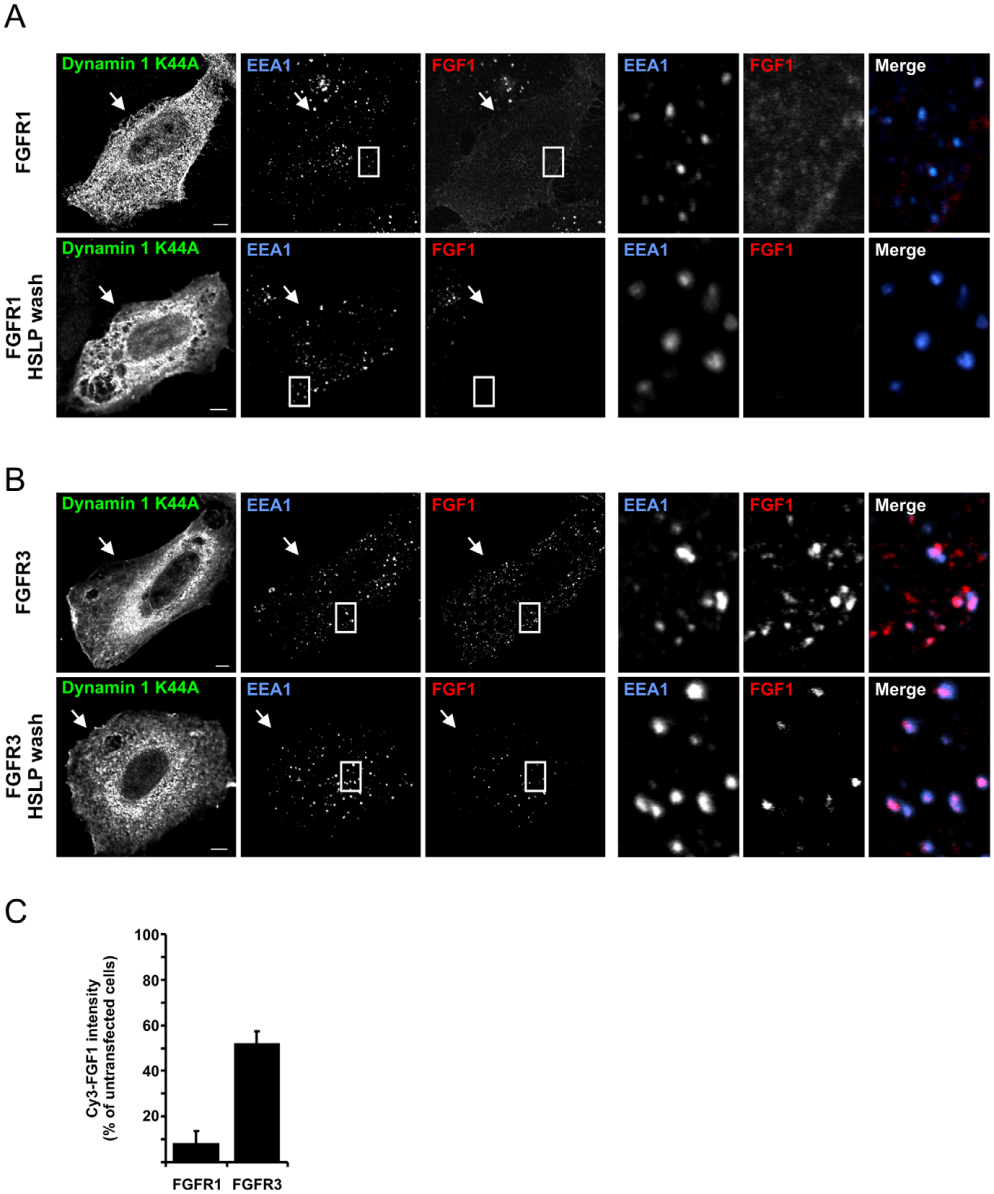
Localization of FGF1 in FGFR1 or FGFR3 cells expressing dynamin 1 K44A. U2OS cells stably transfected with FGFR1 (**A**) or FGFR3 (**B**) were transfected with HA-tagged dynamin 1 K44A and incubated with Cy3-FGF1 and 50 U/ml heparin at 37°C for 20 min. The cells were then fixed and stained with anti-HA and anti-EEA1 antibodies. In some cases the cells were washed with high salt/low pH buffer (HSLP) to remove surface-bound Cy3-FGF1 before fixation. The cells were examined with confocal microscopy. Arrows point to cells transfected with dynamin 1 K44A. Selected areas are enlarged and shown at the right side. Bar, 5 µm. **(C)** The uptake of Cy3-FGF1 was measured as Cy3 intensity in untransfected and dynamin 1 K44A transfected cells. All cells were washed with HSLP-buffer to remove cell-surface bound Cy3-FGF1. The mean intensity of Cy3 in dynamin 1 K44A transfected cells is presented in the histogram as the percentage of Cy3 intensity in untransfected cells. The histogram represents the mean +s.d. of three independent experiments and 20–60 cells were quantified in each case in each experiment. Confocal scanning was performed with identical settings.

Next, we studied if the dots of Cy3-FGF1 in FGFR3 cells expressing the dominant negative dynamin were indeed vesicles containing Cy3-FGF1 or, alternatively, if they were enrichments of Cy3-FGF1 in distinct areas at the cell-surface. For this purpose, FGFR1 and FGFR3 cells transfected with dynamin 1 K44A and incubated with Cy3-FGF1 were either left untreated or washed with high salt, low pH buffer (HSLP) to remove surface bound Cy3-FGF1 [30]. Resistance to HSLP-wash indicates intracellular localization of Cy3-FGF1. The weak Cy3-FGF1 staining in Fig. 4A (upper panel) disappeared upon washing with HSLP-buffer (lower panel) indicating that no Cy3-FGF1 was internalized in FGFR1 cells expressing the dominant negative dynamin. However, in FGFR3 cells expressing the dominant negative dynamin, several Cy3-FGF1 dots were observed (Fig. 4B, upper panel). Even though washing with HSLP clearly reduced the number of Cy3-FGF1 dots, many dots were still visible in the FGFR3 cells upon HSLP-wash (lower panel).

To test if the dots observed in FGFR3, dynamin 1 K44A expressing cells are really intracellular vesicles and not pits at the cell-surface that are protected from the extracellular milieu, the cells were also stained with anti-EEA1 antibody. EEA1 is a protein associated with early/sorting endosomes. Thus, colocalization of Cy3-FGF1 and EEA1 indicates that the Cy3-FGF1 dots are vesicles inside the cell. As expected, in the case of FGFR1 there was no colocalization of the weak Cy3-FGF1 pattern and EEA1 (Fig. 4A). On the other hand, many of the Cy3-FGF1 dots in FGFR3 cells expressing the dominant negative dynamin colocalized with EEA1 in unwashed cells. In HSLP treated cells, there was almost complete colocalization between Cy3-FGF1 and EEA1 (Fig. 4B, see enlarged areas to the right).

These results were further supported by quantifying the intensity of Cy3-FGF1 staining in cells expressing dynamin 1 K44A and comparing it to the intensity of the Cy3-FGF1 staining in nonexpressing cells. The cells were washed with HSLP-buffer before fixation to remove surface-bound Cy3-FGF1. In FGFR1 cells expressing dynamin 1 K44A, the uptake of FGF1 was reduced to about 10% compared to nonexpressing cells. In the FGFR3 cells expressing dynamin 1 K44A the uptake of FGF1 was reduced to only 50% compared to nonexpressing cells (Fig. 4C). Taken together, this indicates that in FGFR3 cells expressing dynamin 1 K44A some of the Cy3-FGF1 is internalized into vesicles whereas some of the Cy3-FGF1 appears to be enriched in distinct areas at the cell surface. Thus, endocytosis of FGF1 via FGFR3 seems to be only partly dependent on dynamin.

### Role of clathrin-independent endocytic regulators in internalization of FGF1 by FGFR1 and FGFR3

**Figure 5:**
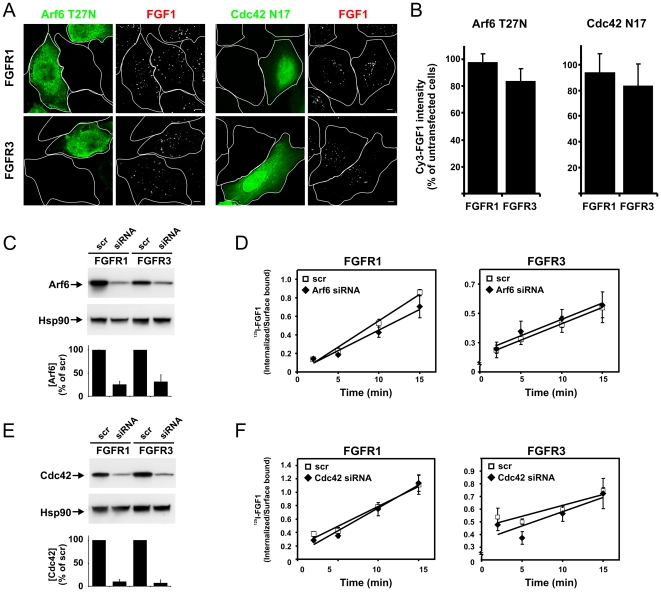
The effect of Arf6 and Cdc42 on endocytosis of FGF1 in cells expressing FGFR1 or FGFR3. (**A**) U2OS cells stably transfected with FGFR1 or FGFR3 were transfected with HA-tagged Arf6 T27N or EGFP-Cdc42 N17 and incubated with Cy3-FGF1 and 50 U/ml heparin at 37°C for 20 min. The cells were then fixed and examined with confocal microscopy. Cells transfected with Arf6 T27N were stained with anti-HA antibody. Bar, 5 µm. (**B**) The uptake of Cy3-FGF1 was measured as Cy3 intensity in untransfected or Arf6 T27N or Cdc42 N17 transfected cells. The mean intensities of Cy3 in transfected cells are presented in the histograms as the percentage of Cy3 intensity in untransfected cells. The histogram represents the mean +s.d. of three independent experiments and 15–55 cells were quantified in each case in each experiment. Confocal scanning was performed with identical settings. U2OS cells stably expressing FGFR1 or FGFR3 were transfected with siRNA oligos (25 nM) targeting Arf6 (**C**) or Cdc42 (**E**) or a non-targeting siRNA control (scr) as described in *Materials and Methods*. The level of knockdown was assessed by Western blotting using rabbit anti-Arf6 antibody or rabbit anti-Cdc42 antibody. Anti-Hsp90 antibody was used as a loading control. One representative Western blot is shown. Western blots were quantified and the bands corresponding to Arf6 and Cdc42 were normalized to loading control and knockdown efficiency presented in the histogram as percentage of non-targeting siRNA control (scr). The histogram represents the mean +s.d. of three independent experiments. U2OS cells stably expressing FGFR1 or FGFR3 were transfected with siRNA oligos (25 nM) targeting Arf6 (**D**) or Cdc42 (**F**) or a non-targeting siRNA control (scr), grown on gelatinized plates and incubated with 10 ng/ml ^125^I-FGF1, 20 U/ml heparin and 0.2% gelatine at 37°C for indicated periods of time. Internalized and surface-bound ^125^I-FGF1 were separated as described in *Materials and Methods* and the ratio was plotted as function of time. Note the different scale on the Y axis. The graph represents the mean ±s.d. of three independent experiments with three parallels.

As Arf6 and Cdc42 have been suggested to regulate two distinct clathrin- and dynamin-independent pathways, we tested whether these GTPases play a role in FGFR3 endocytosis. U2OS cells stably expressing FGFR1 or FGFR3 were transfected with dominant negative mutants of Arf6 (Arf6 T27N) or Cdc42 (Cdc42 N17). The cells were then allowed to endocytose Cy3-FGF1 for 20 minutes before fixation and analysis by confocal microscopy. Cy3-FGF1 was efficiently internalized in transfected and untransfected FGFR1 and FGFR3 cells in both cases (Fig. 5A). Quantification of the intensity of Cy3-FGF1 staining in cells expressing the mutants compared with nonexpressing cells revealed that the Cy3-FGF1 uptake was not significantly changed in either case (Fig. 5B). Also, siRNAs directed against Arf6 or Cdc42 were used to elucidate their role in FGFR3 endocytosis. Despite an apparently efficient knockdown of Arf6 and Cdc42 (Fig. 5C,E and Fig. S4), the rate of endocytosis of ^125^I-FGF1 in depleted FGFR1 or FGFR3 cells was not significantly changed in either case (Fig. 5D,F). Taken together, the data indicate that neither Arf6 nor Cdc42 are major players in FGFR1 or FGFR3 endocytosis in these cells.

In the absence of an effect of perturbed Arf6 or Cdc42 activity in FGFR endocytosis, we chose to test the efficacy of disruption in cell migration assays, since both Cdc42 and Arf6 are known to play important roles in this process [31]–[34]. Using the same conditions as in the endocytosis experiments, we observed that disruption of Cdc42 and Arf6 led to reduced velocity of migrating cells compared to control cells (Fig. S5A,B). This finding indicates that the conditions used above significantly disrupt Arf6 and Cdc42 function.

**Figure 6:**
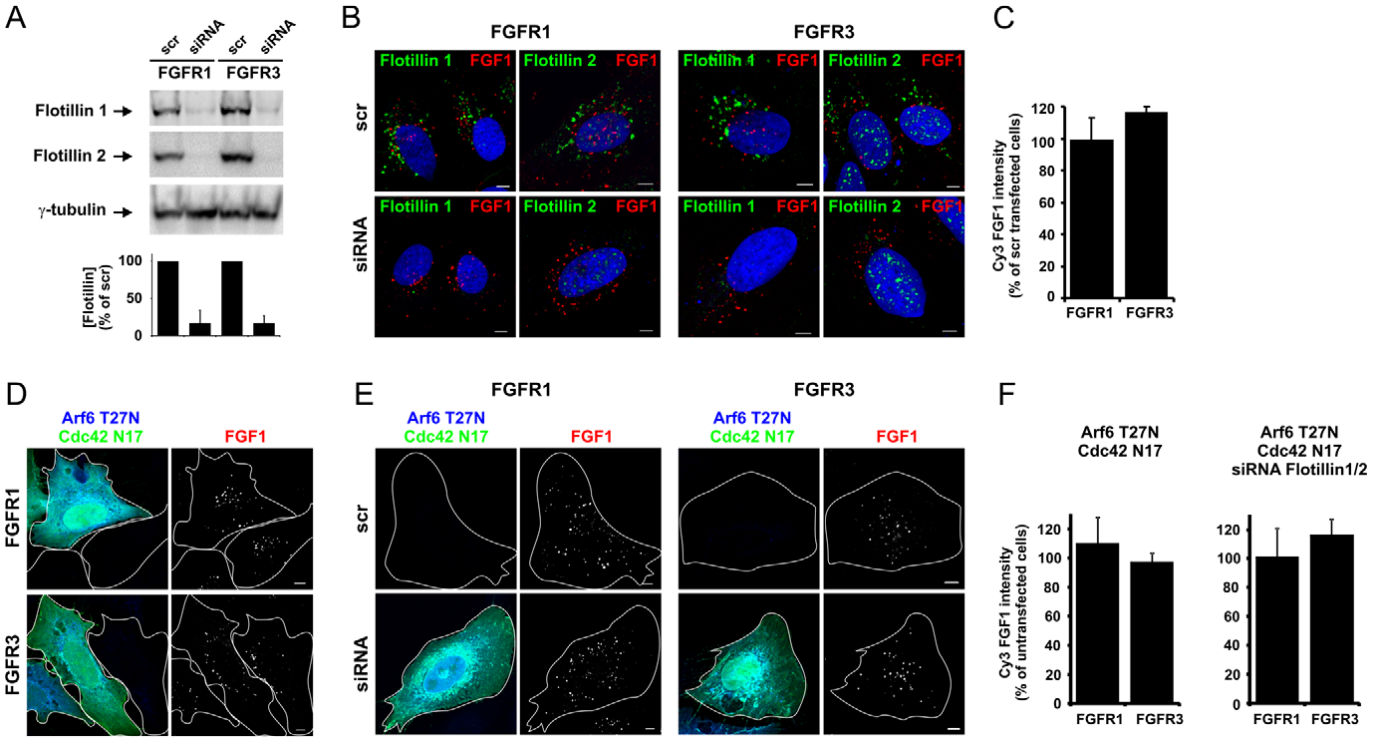
The effect of flotillin 1 and 2 depletion on endocytosis of FGF1 in cells expressing FGFR1 or FGFR3. (**A**) U2OS cells stably expressing FGFR1 or FGFR3 were transfected with siRNA oligos targeting flotillin 1 and flotillin 2 or a non-targeting siRNA control (scr) as described in *Materials and Methods*. The level of knockdown was assessed in every experiment by Western blotting using mouse anti-flotillin 1 and mouse anti-flotillin 2 antibodies. Anti-γ-tubulin antibody was used as a loading control. One representative Western blot is shown. The bands corresponding to flotillin 1 and flotillin 2 were quantified and knockdown efficiency is presented in the histogram as percentage of non-targeting siRNA control (scr). The histogram represents the mean +s.d. of 12 (for flotillin 1) and 7 (for flotillin 2) independent experiments. (**B**) U2OS cells stably expressing FGFR1 or FGFR3 were transfected with siRNA oligos targeting flotillin 1 and 2 or a non-targeting siRNA control (scr). The cells were then incubated for 20 minutes at 37°C with Cy3-FGF1 and 50 U/ml heparin. The cells were stained with rabbit anti-flotillin 1 antibody or rabbit anti-flotillin 2 antibody and examined with confocal microscopy. Bar, 5 µm. (**C**) The uptake of Cy3-FGF1 was measured as Cy3 intensity in non-depleted and flotillin 1 and 2 depleted cells treated as described in B. The mean intensity of Cy3 in flotillin 1 and 2 depleted cells is presented in the histogram as the percentage of Cy3 intensity in non-depleted cells. The histogram represents the mean +s.d. of four independent experiments and 67–138 cells were quantified in each case in each experiment. Confocal scanning was performed with identical settings. (**D**) U2OS cells stably transfected with FGFR1 or FGFR3 were co-transfected with HA-tagged Arf6 T27N and EGFP-Cdc42 N17 and incubated with Cy3-FGF1 and 50 U/ml heparin at 37°C for 20 min. The cells were then fixed, stained with anti-HA antibody and examined with confocal microscopy. Bar, 5 µm. (**E**) U2OS cells stably expressing FGFR1 or FGFR3 were transfected with siRNA oligos targeting flotillin 1 and 2 or a non-targeting siRNA control (scr). The cells were then transfected with HA-tagged Arf6 T27N and EGFP-Cdc42 N17 and incubated for 20 minutes at 37°C with Cy3-FGF1 and 50 U/ml heparin. The cells were fixed, stained with anti-HA antibody and examined with confocal microscopy. Bar, 5 µm. (**F**) The uptake of Cy3-FGF1 was measured as Cy3 intensity in untransfected or co-transfected cells (Arf6 T27N and Cdc42 N17) or in the case of flotillin knockdown as untransfected scr cells or co-transfected (Arf6 T27N and Cdc42 N17), flotillin1/2 siRNA cells. The mean intensities of Cy3 in transfected cells are presented in the histograms as the percentage of Cy3 intensity in untransfected cells. The histogram represents the mean +s.d. of three independent experiments and 32–52 cells were quantified in each case in each experiment. Confocal scanning was performed with identical settings.

Flotillin 1 and 2 (reggie 2 and reggie 1, respectively), have also been implicated in clathrin-independent endocytosis. In order to elucidate their role in FGFR3 endocytosis, siRNA oligos directed against flotillin 1 and 2 were simultaneously transfected into FGFR1 or FGFR3 cells. Knockdown efficiency was examined by Western blotting and qRT-PCR (Fig. 6A and Fig S4). The transfected cells were allowed to endocytose Cy3-FGF1 for 20 minutes and then the cells were fixed, stained with anti-flotillin 1 antibody or anti-flotillin 2 antibody and analysed by confocal microscopy (Fig. 6B). The staining of the flotillins was hardly visible in the cytoplasm in the siRNA transfected cells confirming the efficient knockdown. However, some anti-flotillin 2 antibody staining could be detected in the nucleus of cells even after efficient knockdown of the protein probably representing unspecific antibody-staining. Recently, we performed knockdown of flotillin 1 and 2 using the same oligonucleotides to show their involvement in the retrograde transport of Shiga and Ricin toxin [35]. However, here despite an efficient knockdown, we did not observe any effect on the internalization of Cy3-FGF1. Quantification of the intensity of Cy3-FGF1 staining in cells depleted of flotillins compared to non-depleted cells revealed that the Cy3-FGF1 uptake was not significantly changed in any case (Fig. 6C). Moreover, no colocalization between Cy3-FGF1 and flotillin 1 or 2 were detected. Taken together, the findings indicate that flotillin 1 and 2 are not key players in the endocytic machinery responsible for FGFR1 and FGFR3 internalization in U2OS cells.

It is possible that the clathrin-independent FGFR3 endocytosis could be a product of several pathways. We therefore examined internalization of Cy3-FGF1 upon co-expression of dominant negative Arf6 and Cdc42 (Fig. 6D) and co-expression of dominant negative Arf6 and Cdc42 together with transfection with siRNA directed against Flotillin1 and 2 (Fig. 6E). Internalized Cy3-FGF1 was observed in any case and quantification of the intensity of the Cy3-FGF1 staining revealed that the Cy3-FGF1 uptake was not significantly changed compared to control cells (Fig. 6F). Taken together the data indicate that FGFR3 does not utilize any of the well-known clathrin-and dynamin-independent endocytic pathways that have been described to date.

### Signalling from FGFR1 and FGFR3 upon disruption of endocytosis

**Figure 7:**
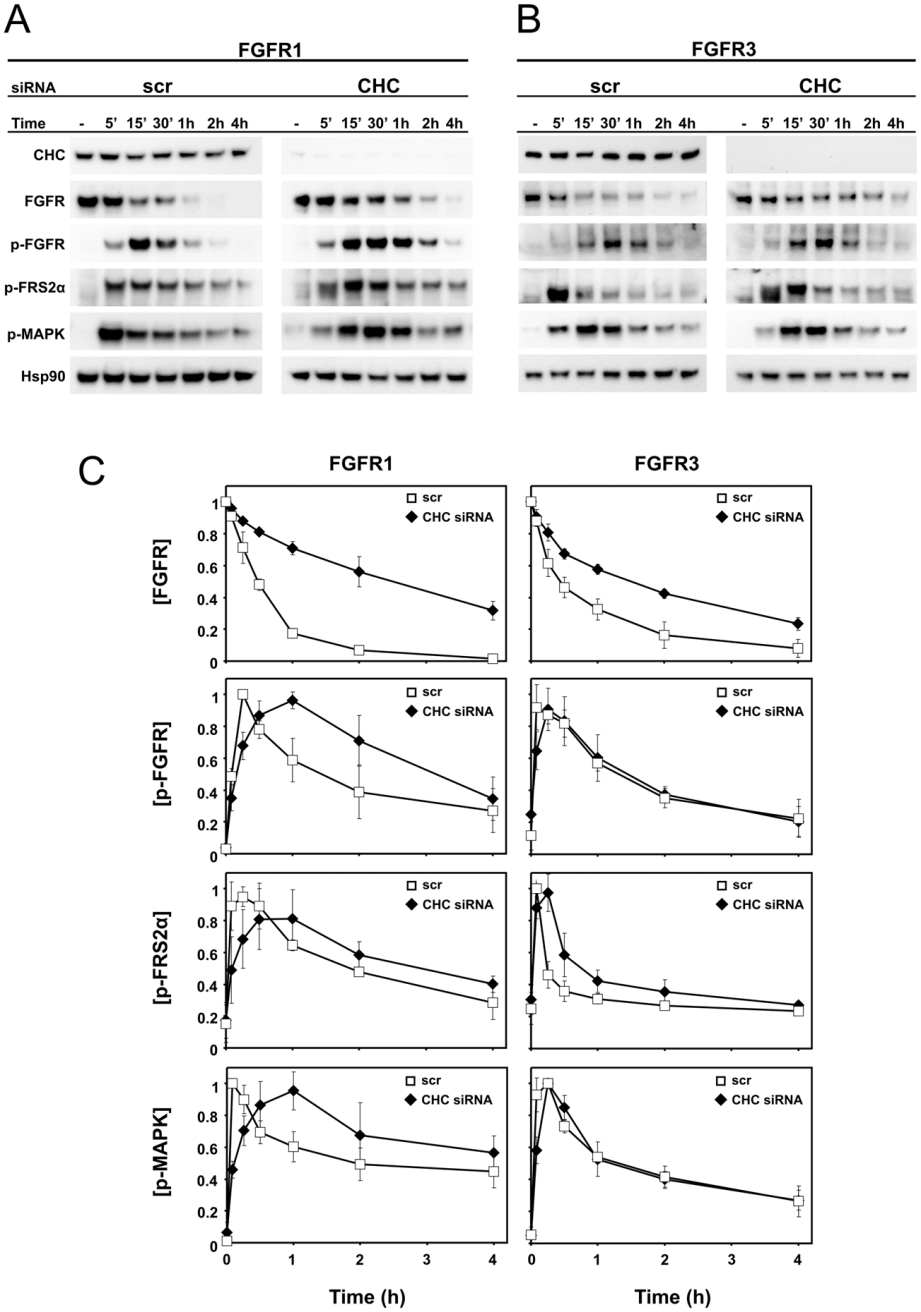
Degradation and signalling in FGFR1 and FGFR3 cells upon clathrin heavy chain depletion. U2OS cells stably expressing FGFR1 (**A**) or FGFR3 (**B**) were transfected with siRNA oligos (100 nM) targeting CHC or a non-targeting siRNA control (scr), serum-starved for 4–6 hours and then stimulated with the growth factor in the presence of heparin (20 U/ml) and cycloheximide (10 µg/ml) for different time points. Cells were lysed, and the cellular material was analyzed by SDS-PAGE and immunoblotting using following antibodies: anti CHC, anti-FGFR1/anti-FGFR3, anti-phospho-FGFR (p-FGFR), anti-phospho-FRS2α (p-FRS2α), anti-phospho-MAPK (p-MAPK) and anti-Hsp90 as a loading control. (**C**) The amount of FGFR, p-FGFR, p-FRS2α and p-MAPK were expressed as a percentage with the maximum for each protein set to 1. The graph represents the mean ±s.d. of three independent experiments.

To examine how endocytosis controls FGFR signalling, we studied FGFR turnover and signalling in FGFR1 and FGFR3 cells that have been depleted of CHC by oligo-based siRNA. The cells were serum-starved for 4 to 6 hours and then stimulated with FGF1 for different periods of time in the presence of cycloheximide. Cycloheximide was added to prevent the appearance of newly synthesized receptors. The cells were then lysed and subjected to immunoblotting with indicated antibodies followed by quantification (Fig. 7A,B,C).

As seen in Fig. 7A and C, the bands corresponding to FGFR1 in CHC depleted cells were more stable over time than those corresponding to FGFR1 in non-depleted cells. This indicates that FGFR1 is degraded more slowly in cells depleted of clathrin. Also the bands corresponding to phosphorylated FGFR1 and the phosphorylated downstream effectors such as FRS2α (fibroblast growth factor receptor substrate 2α) and MAPK p42/p44 were more stable over time in clathrin-depleted cells than in non-depleted cells. Taken together, these results indicate that FGFR1 signalling is prolonged upon CHC depletion due to decreased degradation of FGFR1. In FGFR3 cells, the differences between scrambled siRNA transfected and CHC siRNA transfected cells were not as pronounced as in the case of FGFR1 (Fig. 7B,C). The bands corresponding to FGFR3 in cells depleted of clathrin were somewhat stronger over time than in non-depleted cells.

Interestingly, the intensities of the bands corresponding to phosphorylated FGFR1, FRS2α and MAPK p42/44 peaked at later time points in clathrin-depleted FGFR1 cells compared to non-depleted FGFR1 cells. For example, the intensities of the bands corresponding to phosphorylated MAPK p42/44 peaked 30–60 minutes after addition of FGF1 in CHC-depleted FGFR1 cells whereas in non-depleted FGFR1 cells the bands corresponding to phosphorylated MAPK p42/44 peaked already 5 minutes after addition of FGF1. In the case of FGFR3, the bands corresponding to phosphorylated FRS2α and MAPK p42/44 were only slightly stronger at earlier time points in non-depleted cells than in CHC-depleted cells. This is in accordance with the observation that endocytosis of FGFR3 is only partly dependent on clathrin. These results indicate that clathrin-mediated endocytosis is required for efficient activation of certain FGFR1 downstream signalling molecules, but not for FGFR3.

## Discussion

Internalization of signalling receptors from the cell surface is the first step in a series of events that influence the signalling output. We have here demonstrated that internalization of FGFR1 and FGFR3 is dependent on different molecular machineries. Internalization of FGFR1 was severely reduced when cells were depleted of clathrin or by expression of a dominant negative mutant of dynamin. This indicates that when bound to FGFR1, FGF1 is mainly endocytosed through clathrin mediated endocytosis. Internalization of FGFR3 was only partly inhibited by clathrin depletion. Expression of a dominant negative construct of dynamin induced accumulation of FGFR3 in defined areas at the cell surface and reduced, but did not abolish, the amount of internalized receptor. These results suggest that FGFR3 is internalized via both clathrin-dependent and clathrin-independent endocytosis. Depletion of clathrin decreased the degradation of FGFR1 due to delayed transport to the lysosomes. In contrast, FGFR3 degradation was only slightly delayed upon depletion of clathrin. Moreover, full, immediate activation of FGFR1 signalling required clathrin-mediated endocytosis whereas activation of FGFR3 signalling was only slightly delayed upon clathrin-depletion. As FGFR3 is endocytosed with a lower rate than FGFR1, it implies that the clathrin-independent endocytic component of FGFR3 internalization is a slower process than the classic clathrin-mediated endocytosis.

It is not clear which molecules are mediating the clathrin-independent endocytosis of FGFR3. According to the classification of non-clathrin endocytosis [8], [9] the Cdc42 (GEEK/CLIC)-, the flotillin- and the Arf6-mediated pathways are independent of dynamin. We therefore examined whether interfering with any of these proteins affected FGFR endocytosis. However, the uptake of FGF1 in FGFR3 or FGFR1 cells was not significantly altered upon expressing of dominant negative mutants or depletion of cells of these proteins. Arf6, Cdc42 and flotillin seem not to be major players in FGFR3 endocytosis in the U2OS cells. Probably, other clathrin- and dynamin independent pathways exist [10], [12] and FGFR3 may be a cargo protein in such a new uncharacterized endocytic pathway.

It should be kept in mind that interfering with the endocytic machinery may activate new pathways or cause upregulation of such ones that normally play only a minor role [36]. Here, we have compared endocytosis of FGFR1 and FGFR3 in the same cellular system and under the same conditions using U2OS cells stably transfected with either receptor. It is likely that the upregulation or activation of other endocytic pathways would be similar in both cases and thus, that the observed clathrin-independent endocytosis of FGFR3 is not due to such effects. It should also be noted that the rate of FGFR1 endocytosis during clathrin knockdown is similar to that of FGFR3. It can therefore not be excluded that a minor fraction of FGFR1 could also be endocytosed through a clathrin independent mechanism.

Although we analyzed several independent clones of stably transfected U2OS cells, the FGFR1 cells generally provided about three times as many cell surface binding-sites for FGF1 as the FGFR3 cells (Fig. S3). The amount of the receptors at the cell surface may influence the internalization in several ways. Firstly, a larger number of receptors at the cell surface may induce more clathrin-independent internalization of receptors due to saturation of the clathrin-dependent endocytic system [37]. Even though FGFR1 was overexpressed in the U2OS cells, FGFR1 seems to be mainly internalized by clathrin-mediated endocytosis. Since FGFR1 was expressed to a higher extent than FGFR3, it is not likely that the observed clathrin-independent uptake of FGFR3 was due to overexpression and constitutive internalization of the receptor. Secondly, it has been suggested that high concentrations of ligand might direct the receptors to clathrin independent endocytosis whereas low concentrations of ligand direct the receptor to clathrin-mediated endocytosis [13], [27]. However, we have tested ligand concentrations from 2–100 ng/ml and clathrin-independent uptake of FGFR3 was observed in all cases.

Depletion of clathrin decreased the degradation of FGFR1, and prolonged its signalling properties. This probably reflects the significant role internalization plays on receptor signalling. Clathrin might also play a role in endosomal sorting and recycling [38], [39]. It is possible that the signalling from the receptors is prolonged upon clathrin heavy chain depletion due to both inefficient internalization and inefficient sorting to the lysosomes. However, consistent with the observation that FGFR3 is internalized via both clathrin-dependent and clathrin-independent endocytosis, the signalling through FGFR3 upon clathrin depletion was much less prolonged than the signalling through FGFR1.

It has been suggested that receptors in endosomes can recruit and activate other downstream signalling molecules than receptors at the cell surface [1]. Here, we report prolonged signalling from receptors that are trapped at the cell surface due to clathrin depletion. However, full phosphorylation of FGFR and of downstream signalling molecules was reached at a later time point in clathrin depleted cells than in non-depleted cells. This indicates that endocytic trafficking is required for full activation of the signalling pathways here examined.

The 22 FGFs and 4 FGFRs make up a large and complex signalling system with the ability to mediate a wide spectrum of biological effects in different types of cells. However, the various signalling complexes mainly activate the same intracellular signalling pathways. Spatio-temporal regulation of FGFR signalling adds diversity to the signalling system and can generate different signalling output. We have here reported that FGFR1 and FGFR3 are endocytosed via different mechanisms and thus are dependent on different endocytic machineries which can regulate both the duration and the strength of their signalling. This introduces additional diversity into the FGFR signalling system.

Knowledge of the mechanisms of internalization is important to understand how signalling receptors and other molecules in the plasma membrane are regulated. In the case of FGFRs such knowledge might open up for development of treatments for disorders associated with excess FGFR signalling.

## Materials and Methods

### Antibodies and reagents

The following primary antibodies were used: mouse anti-HA.11 (Covance, Nordic Biosite, Täby, Sweden); mouse anti-early endosomal antigen (EEA) 1, mouse anti-Hsp90, mouse anti-flotillin 1, mouse anti-flotillin 2 (BD Biosciences Transduction Laboratories, Lexington, KY); mouse anti-CHC (RDI division of Fitzgerald Industries, Concorde, MA); goat anti-CHC, rabbit anti-FGFR1, rabbit anti-FGFR3 (Santa Cruz Biotechnology, Santa Cruz, CA); rabbit anti-HA, mouse anti-Myc Tag, clone 4A6 (Millipore, Billerica, MA); mouse anti-phospho-FGFR, rabbit anti-phospho-FRS2α (Y196), rabbit anti-MAPK (p42/p44), mouse anti-phospho-MAPK (p42/p44), rabbit anti-Cdc42, rabbit anti-Arf6 (Cell Signaling Technology, Danvers, MA); rabbit anti-flotillin-2, mouse anti-γ-tubulin (Sigma-Aldrich, St.Louis, MO). Rabbit anti-Arf6 was a generous gift from Dr. J.G. Donaldson (NIH, Bethesda, MD) and rabbit anti-flotillin-1 was a generous gift from Dr. G. van der Goot (EPFL, Lausanne, Switzerland). Secondary antibodies were from Jackson Immuno-Research Laboratories (West Grove, PA). Alexa 647-Tf was from Invitrogen (Carlsbad, CA). FGF1 was labelled with Cy3-maleimide (GE Healthcare, Chalfont St. Giles, United Kingdom) following the manufacturer's procedures. FGF1 was iodinated by the Iodogen method according to the manufacturer's protocol (Pierce Chemical, Rockford, IL). [^125^I]Na was from PerkinElmer. The following reagents were used: cycloheximide, 10% formalin solution (approximately 4% formaldehyde), heparin, Pronase E (Sigma-Aldrich); DharmaFECT transfection reagent 1 and 2 (Dharmacon RNA Technologies, Lafayette, CO); DOTAP, Fugene 6 (Roche Diagnostics, Indianapolis, IN); Geneticin (G-418), Lipofectamine RNAiMAX transfection reagent, Hoechst 33342 (Invitrogen); Mowiol (Calbiochem, San Diego, CA); DRAQ5 (Biostatus Limited, Leicestershire, United Kingdom); RNeasy plus mini kit, QuantiTect SYBR green PCR kit, QuantiTect primers (Qiagen); iScript cDNA synthesis kit (Bio-Rad Laboratories), MatTek 35 mm glass-bottom dishes (MatTek corporations, Ashland, MA). ^125^I-Tf was a generous gift from Anne Grete Myrann, this institute.

### Plasmids and siRNA oligos

pcDNA3-hFGFR1, pcDNA3-hFGFR2 and pcDNA3-hFGFR4 has been described previously [25]. pcDNA3-hFGFR3 was a generous gift from Dr. A. Yayon (ProChon Biotech, Ness Ziona, Israel). pcDNA3.1 hemagglutinin epitope (HA) tagged wild-type and K44A mutant constructs of dynamin 1 and dynamin 2 were a generous gift from Dr. S. L. Schmid (The Scripps Research Institute, La Jolla, CA). pXS HA tagged Arf6 T27N [40] was a generous gift from Dr. J. G. Donaldson (NIH, Bethesda, MD). pEGFP-C1-Cdc42 N17 [41] was a generous gift from Dr. F. Sanchez-Madrid (Universidad Autonoma de Madrid, Madrid, Spain). Vector-based siRNA against CHC has been described previously [42]. siRNA oligos targeting CHC (targeting sequence: 5-GCAATGAGCTGTTTGAAGA-3) and siRNA targeting Arf6 (targeting sequence: 5-AAGGTCTCATCTTCGTAGTGG-3) were purchased from MWG Biotech (Ebersberg, Germany) and have been described previously [6], [43]. siRNA oligos targeting flotillin 1 (targeting sequence: 5-GCAGAGAAGUCCCAACUAAUU-3), flotillin 2 (targeting sequence: 5-GAGGUUGUGCAGCGCAAUU-3) Cdc42 (targeting sequence: 5-CTCCTGATATCCTACACAA-3), and ON-TARGETplus siCONTROL siRNA were purchased from Dharmacon RNA Technologies. The siRNA oligos targeting flotillin 1 and 2 have been described previously [35].

### Cells and transfection

Transient expression of the different constructs was performed by transfecting cells with the plasmid DNA using Fugene 6 transfection reagent according to the manufacturer's protocol. Cells were seeded into plates the day preceding the transfection and experiments were performed 16–24 hours after transfection. For the vector-based knockdown of clathrin, the cells were transfected with vector-based siRNA against CHC for 96 hours. Oligo-based knockdown was performed using DharmaFECT transfection reagent or Lipofectamine RNAiMAX transfection reagent according to the manufacturer's protocol. Experiments were performed 48 hours (Arf6 and Cdc42 siRNA) or 72 hours (CHC siRNA and flotillin 1 and 2) after transfection. DOTAP liposomal transfection reagent was used according to the manufacturer's protocol to obtain U2OS cells stably expressing FGFR3. Clones were selected with 1 mg/ml geneticin. Clones were chosen based on their receptor expression level analyzed by immunofluorescence and immunoblotting. U2OS cells stably expressing FGFR1 have been described previously [20]. The cells were propagated in DMEM, supplemented with 10% fetal bovine serum, 100 U/ml penicillin and 100 µg/ml streptomycin in a 5% CO_2_ atmosphere at 37°C. In addition, 0.2–1 mg/ml geneticin was added to the growth media of stably transfected U2OS cells.

### RNA isolation, cDNA synthesis and quantitative real-time polymerase reaction (qRT-PCR)

Total RNA was isolated from cell lysate using RNeasy plus mini kit and the QIAcube robot (Qiagen) according to the manufacturer's protocol. Then 0.5 µg of RNA was used for cDNA synthesis using iScript cDNA synthesis kit. Quantitative real-time PCR was performed using QuantiTect SYBR Green PCR kit, cDNA template and the following QuantiTect primers: Arf6 (QT01681582), Cdc42 (QT01674442), Flotillin 1 (QT00036743), Flotillin 2 (QT00079926) and Succinate dehydrogenase (SDHA) (QT00059486). The qRT-PCR was run and analysed using the Lightcycler 480 (Roche). Cycling conditions were 5 minutes at 95°C followed by 45 cycles 10 seconds at 95°C, 20 seconds at 60°C and 10 seconds at 72°C. Gene amplification was normalized to the expression of SDHA.

### Laser scanning confocal microscopy

Cells grown on coverslips were incubated with 100 ng/ml Cy3-FGF1 for 20 minutes at 37°C in the presence of 50 U/ml heparin. The cells were fixed in 4% formaldehyde solution and mounted in Mowiol. In some cases the cells were washed with high salt/low pH (HSLP) buffer (2 M NaCl and 20 mM NaAc, pH 4.0) before fixation. In some experiments the cells were in addition to Cy3-FGF1 incubated with 5 µg/ml Alexa 647-Tf. When antibodies were used to visualize structures within the cell, the cells were permeabilized with 0.1% Triton X-100 and incubated with the primary antibody for 20 minutes, washed and then incubated with the secondary antibody coupled to a fluorophore for 20 minutes before mounting in Mowiol. The cells were examined with a Zeiss LSM 510 META confocal microscope (Carl Zeiss, Jena, Germany). Images were prepared with Zeiss LSM Image Browser version 3.2 (Carl Zeiss) and CorelDRAW11 (Corel, Fremont, CA). For quantification of Cy3-FGF1 uptake, cells randomly located on the coverslips were scanned at fixed intensity settings below pixel-saturation and the total cellular intensity was determined using the histogram function in the Zeiss LSM 510 Software (Carl Zeiss). All pixel values above the background level were quantified.

### Internalization of ^125^I-FGF1

Internalization experiments were performed on confluent cells growing on 12-well gelatinized microtiter plates incubated for indicated periods of time at 37°C with indicated concentrations of ^125^I-FGF1 in HEPES medium containing 0.2% gelatine and 20 U/ml heparin. The cells were then washed twice with ice-cold HEPES medium and once in washing buffer (1.14 M NaCl and 10 mM NaH_2_PO_4_, pH 7.4). Surface bound ^125^I-FGF1 was collected after removal with high salt/low pH (HSLP) buffer and finally, internalized ^125^I-FGF1 was collected after solubilisation of cells in 1M KOH. Radioactivity was measured with a γ-counter and the ratio of internalized to surface-localized ^125^I-FGF1 was plotted as function of time.

### ^125^I-FGF1 binding experiments

^125^I-FGF1 saturation binding experiments were essentially performed as previously published experiments [30]. The cells were incubated for 2 hours at 4°C in HEPES medium containing 20 U/ml heparin, 0.2% gelatine and increasing concentrations of ^125^I-FGF1. Then, the cells were washed twice with ice-cold HEPES and once in 1 M NaCl in PBS. Cells were solubilised in 1 M KOH and the solubilised radioactivity was measured with a γ-counter.

### Internalization of ^125^I-Tf

Internalization experiments were performed on confluent cells growing on 12-well gelatinized microtiter plates incubated for 2 minutes at 37°C with ^125^I-Tf in HEPES medium containing 0.2% gelatine. The cells were then washed three times with ice-cold HEPES medium and incubated 1 hour at 4°C with HEPES medium containing 2 mg/ml pronase. After pronase treatment the medium containing the cells was centrifuged for 2 minutes before the radioactivity in the cell pellet (endocytosed) and in the supernatant (surface bound) were measured with a γ-counter. Endocytosed ^125^I-Tf was calculated as the percentage of total cell-associated (endocytosed and surface-bound) transferrin.

### Western blot analysis of FGFR degradation

Cells transfected with CHC siRNA were serum-starved for 4–6 hours and then stimulated with 30 ng/ml FGF1 in the presence of 10 U/ml heparin and 10 µg/ml cycloheximide for different time points. The cells were lysed with SDS sample buffer, scraped and sonicated. Total cell lysates were separated by SDS-PAGE, transferred onto Immobilon-P membrane and subjected to immunoblot analysis. The membrane was stripped and re-probed with different antibodies. ImageQuant version 5 was used for quantification of the intensity of the bands of interest.

### Cell migration analysis

Cells were plated on 35 mm glass-bottom MatTek dishes, transfected as described above and then observed for a period of 9–15 hours using a Biostation IM (Nikon, Melville, NY). Cells were maintained in a 5% CO_2_ atmosphere at 37°C throughout the observation period. Pictures of the cells were acquired every 10 minutes and velocity of migration was determined using the Image J software.

## Supporting Information

Figure S1 **Examination of level of endogenous FGFRs in U2OS cells.** Untransfected U2OS cells (U2OS) or U2OS cells stably expressing FGFR1 or FGFR3 (U2OS FGFR1/U2OS FGFR3) or U2OS cells transiently transfected with FGFR2 or FGFR4 (FGFR2/FGFR4) were lysed and the cellular material was analyzed by SDS-PAGE and immunoblotting using the indicated antibody.
(TIF)

Figure S2 (**A**) **Ability of FGF1 to bind to cells depleted of clathrin heavy chain.** Binding of ^125^I-FGF1 (∼28 000 cpm/ng) to U2OS cells stably expressing FGFR1 or FGFR3 and depleted of CHC by siRNA oligo-mediated knockdown was measured by adding increasing concentrations of the labelled growth factor to the cells at 4°C in the presence of 20 U/ml heparin and 0.2% gelatine. After 2 hours unbound ^125^I-FGF1 was removed by washing and the amount of radioactivity associated with the cells was measured. The graph represents the mean ±s.d. of three independent experiments with three parallels. (**B**) **The effect of clathrin heavy chain siRNA on endocytosis of Tf in cells expressing FGFR1 or FGFR3.** U2OS cells stably expressing FGFR1 or FGFR3 were transfected with siRNA oligos targeting CHC or a non-targeting siRNA control (scr), grown on gelatinized plates and incubated with ^125^I-Tf and 0.2% gelatine at 37°C for 2 minutes. Internalized and surface-bound ^125^I-Tf were separated as described in *Materials and methods* and endocytosed ^125^I-Tf is presented as percentage of total cell associated. The graph represents one independent experiment with three parallels +s.d.
(TIF)

Figure S3 **Ability of FGF1 to bind to cells stably expressing FGFR1 or FGFR3.** Binding of ^125^I-FGF1 (∼20 000 cpm/ng) to U2OS cells stably expressing FGFR1 or FGFR3 was measured by adding increasing concentrations of the labelled growth factor to the cells (approx. 300 000 cells/well) at 4°C in the presence of 20 U/ml heparin and 0.2% gelatine. After 2 hours unbound ^125^I-FGF1 was removed by washing and the amount of radioactivity associated with the cells was measured. The number of binding sites was calculated based on the presumption that saturation was achieved at 25 ng/ml FGF1. The graph represents the mean ±s.d. of two independent experiments with three parallels.
(TIF)

Figure S4 **Knockdown efficiency examined by mRNA level.** RNA isolation, cDNA synthesis and qRT-PCR were performed as described in materials and methods. The amount of mRNA were calculated relative to the housekeeping gene SDHA and are expressed as percentage of scr. The histograms represent the mean +s.d. of two independent experiments.
(TIF)

Figure S5 **Validation of disruption of Arf6 and Cdc42.** (**A**) U2OS cells stably expressing FGFR3 were transfected with siRNA oligos targeting Arf6 or a non-targeting siRNA control (scr) and monitored by imaging every 10 minutes for 9 hours in the presence of 100 ng/ml FGF1 and 20 U/ml heparin. The velocity of migration was quantified and the mean velocity is presented in the histogram. The histogram represents one independent experiment +s.d. and 39–40 cells were quantified for each condition. (**B**) U2OS cells stably expressing FGFR3 were transfected with EGFP-Cdc42 N17 and cell migration was monitored by imaging every 10 minutes for 15 hours in the presence of 100 ng/ml FGF1 and 20 U/ml heparin. The velocity of migration was quantified and the mean velocity is presented in the histogram. The histogram represents one independent experiment +s.d. and 10 cells were quantified for each condition.
(TIF)
