# Supplementary figures and images for: Clathrin- and Dynamin-Independent Endocytosis of FGFR3 – Implications for Signalling
Source: PLoS One. 2011 Jul 14;6(7):e21708. doi: 10.1371/journal.pone.0021708 (PMC3136467; doi:10.1371/journal.pone.0021708)

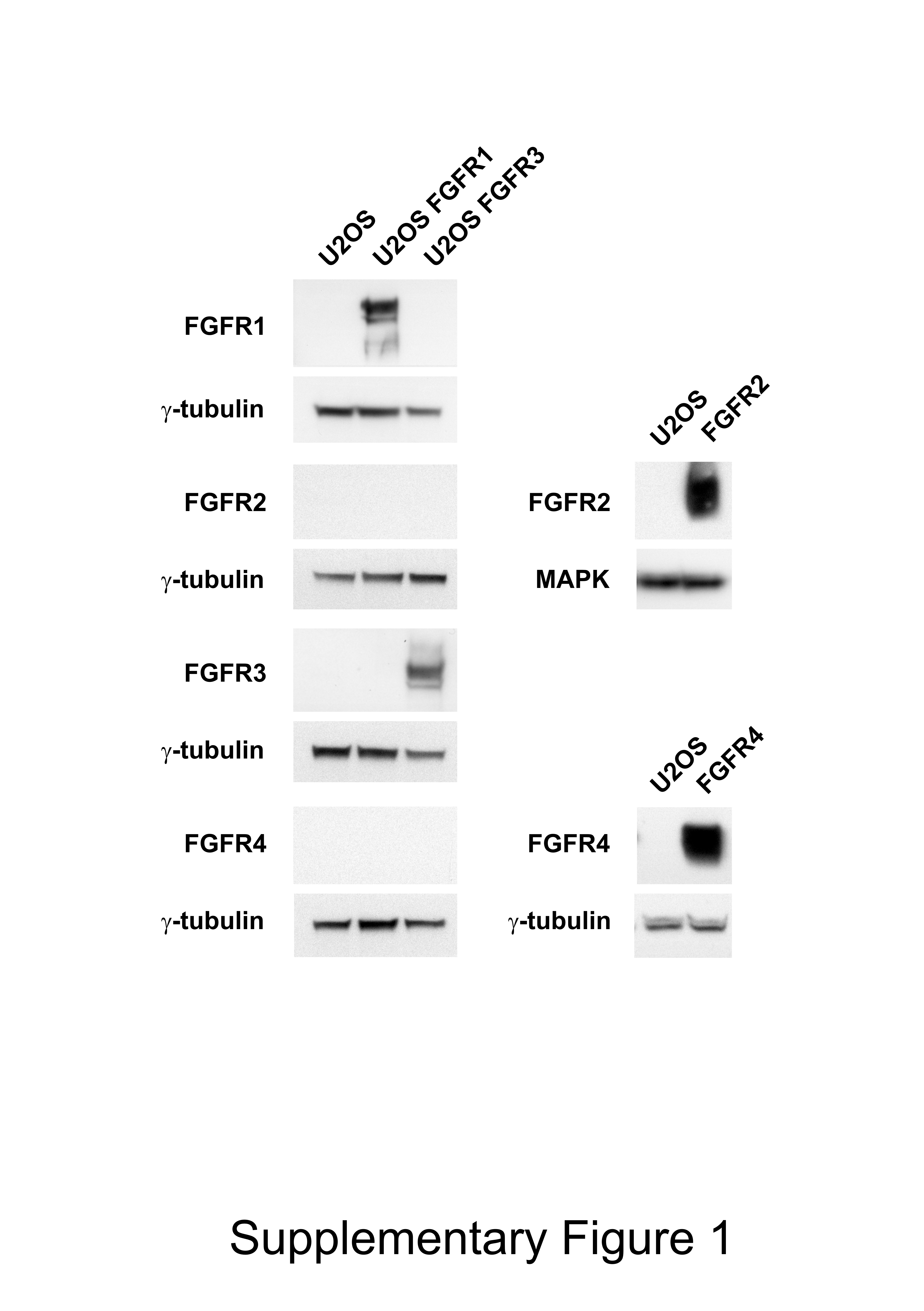

Supplement: Figure S1 — Examination of level of endogenous FGFRs in U2OS cells. Untransfected U2OS cells (U2OS) or U2OS cells stably expressing FGFR1 or FGFR3 (U2OS FGFR1/U2OS FGFR3) or U2OS cells transiently transfected with FGFR2 or FGFR4 (FGFR2/FGFR4) were lysed and the cellular material was analyzed by SDS-PAGE and immunoblotting using the indicated antibody. (TIF) [file pone.0021708.s001.tif]

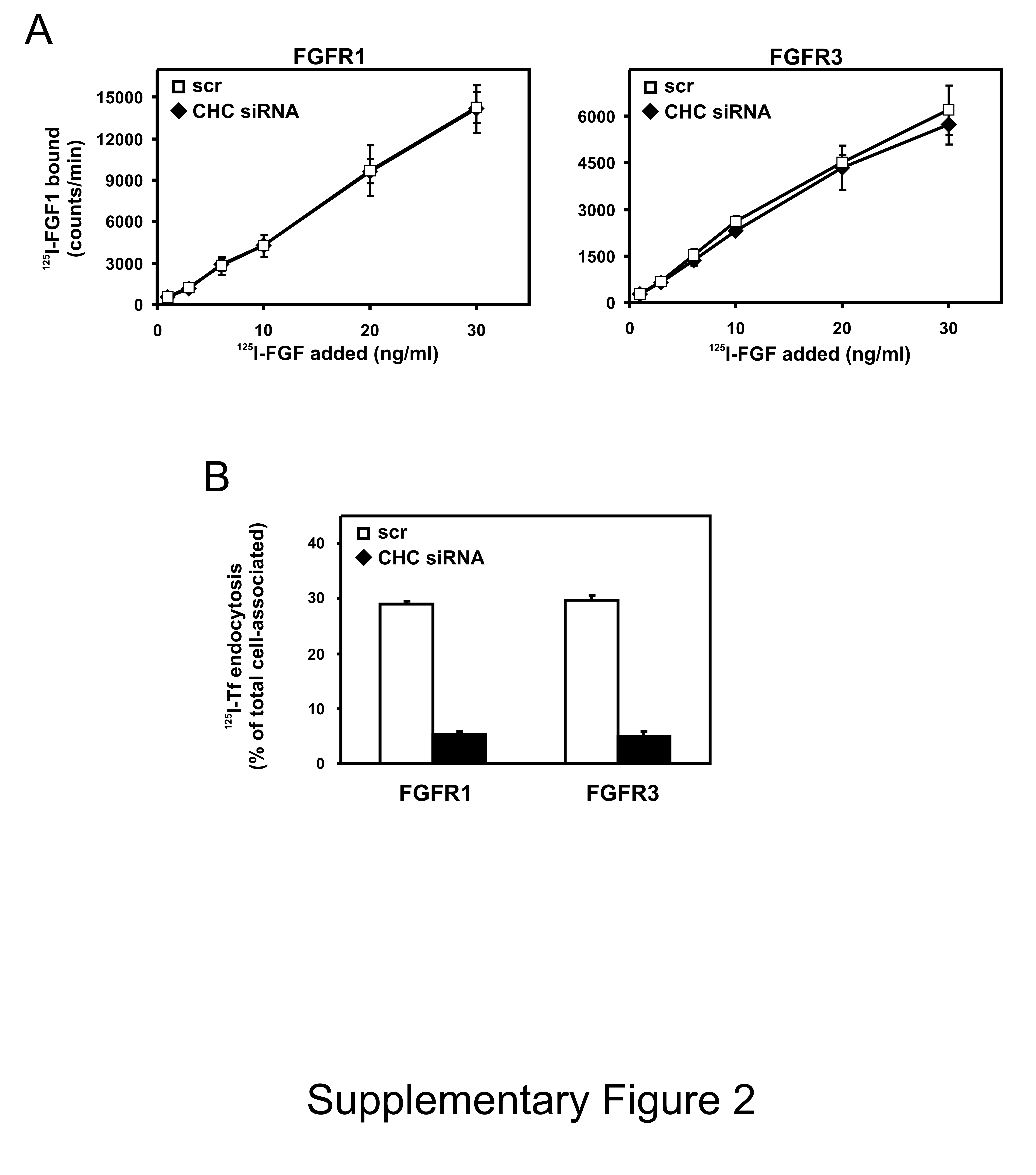

Supplement: Figure S2 — (A) Ability of FGF1 to bind to cells depleted of clathrin heavy chain. Binding of 125I-FGF1 (∼28 000 cpm/ng) to U2OS cells stably expressing FGFR1 or FGFR3 and depleted of CHC by siRNA oligo-mediated knockdown was measured by adding increasing concentrations of the labelled growth factor to the cells at 4°C in the presence of 20 U/ml heparin and 0.2% gelatine. After 2 hours unbound 125I-FGF1 was removed by washing and the amount of radioactivity associated with the cells was measured. The graph represents the mean ±s.d. of three independent experiments with three parallels. (B) The effect of clathrin heavy chain siRNA on endocytosis of Tf in cells expressing FGFR1 or FGFR3. U2OS cells stably expressing FGFR1 or FGFR3 were transfected with siRNA oligos targeting CHC or a non-targeting siRNA control (scr), grown on gelatinized plates and incubated with 125I-Tf and 0.2% gelatine at 37°C for 2 minutes. Internalized and surface-bound 125I-Tf were separated as described in Materials and methods and endocytosed 125I-Tf is presented as percentage of total cell associated. The graph represents one independent experiment with three parallels +s.d. (TIF) [file pone.0021708.s002.tif]

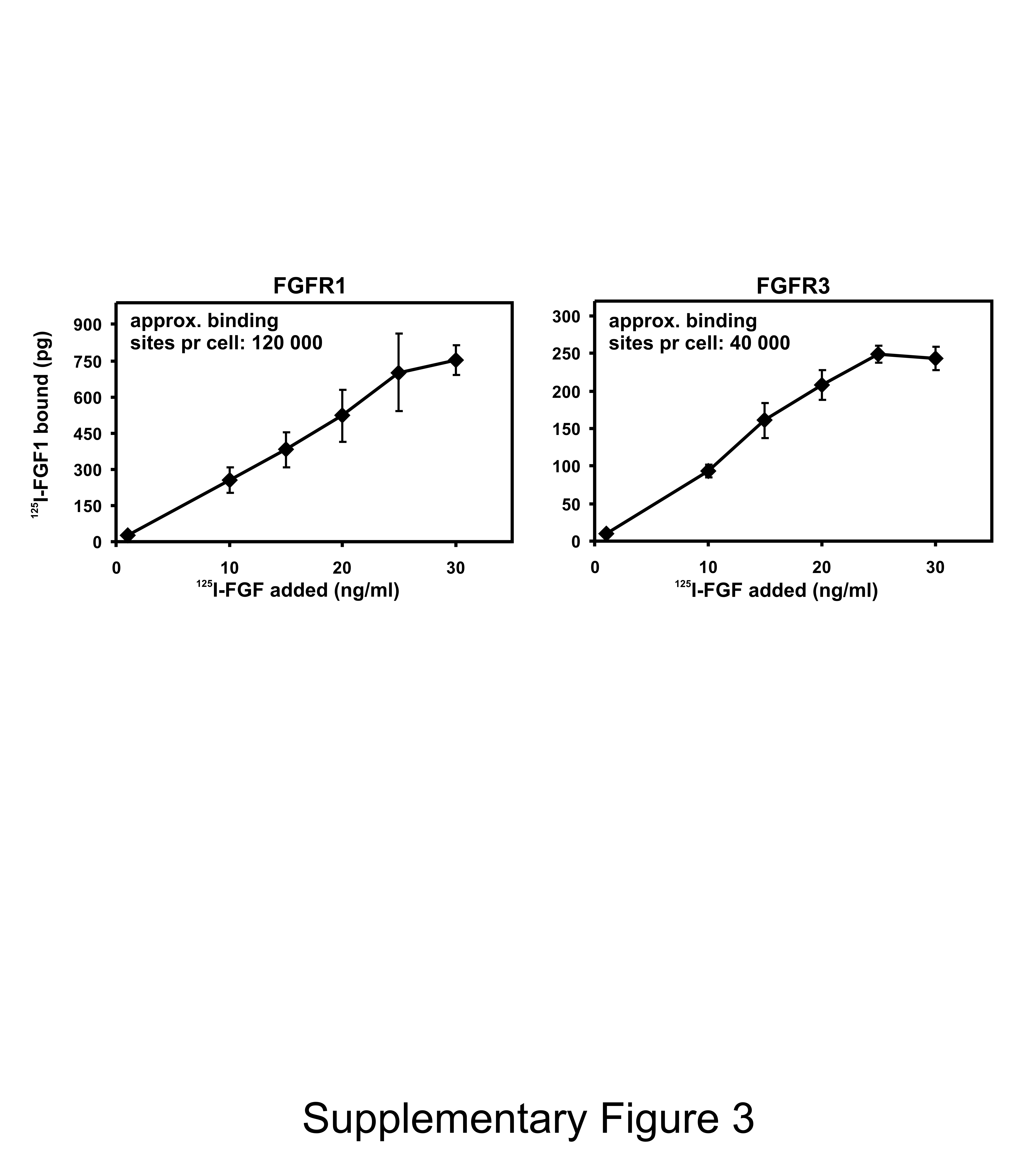

Supplement: Figure S3 — Ability of FGF1 to bind to cells stably expressing FGFR1 or FGFR3. Binding of 125I-FGF1 (∼20 000 cpm/ng) to U2OS cells stably expressing FGFR1 or FGFR3 was measured by adding increasing concentrations of the labelled growth factor to the cells (approx. 300 000 cells/well) at 4°C in the presence of 20 U/ml heparin and 0.2% gelatine. After 2 hours unbound 125I-FGF1 was removed by washing and the amount of radioactivity associated with the cells was measured. The number of binding sites was calculated based on the presumption that saturation was achieved at 25 ng/ml FGF1. The graph represents the mean ±s.d. of two independent experiments with three parallels. (TIF) [file pone.0021708.s003.tif]

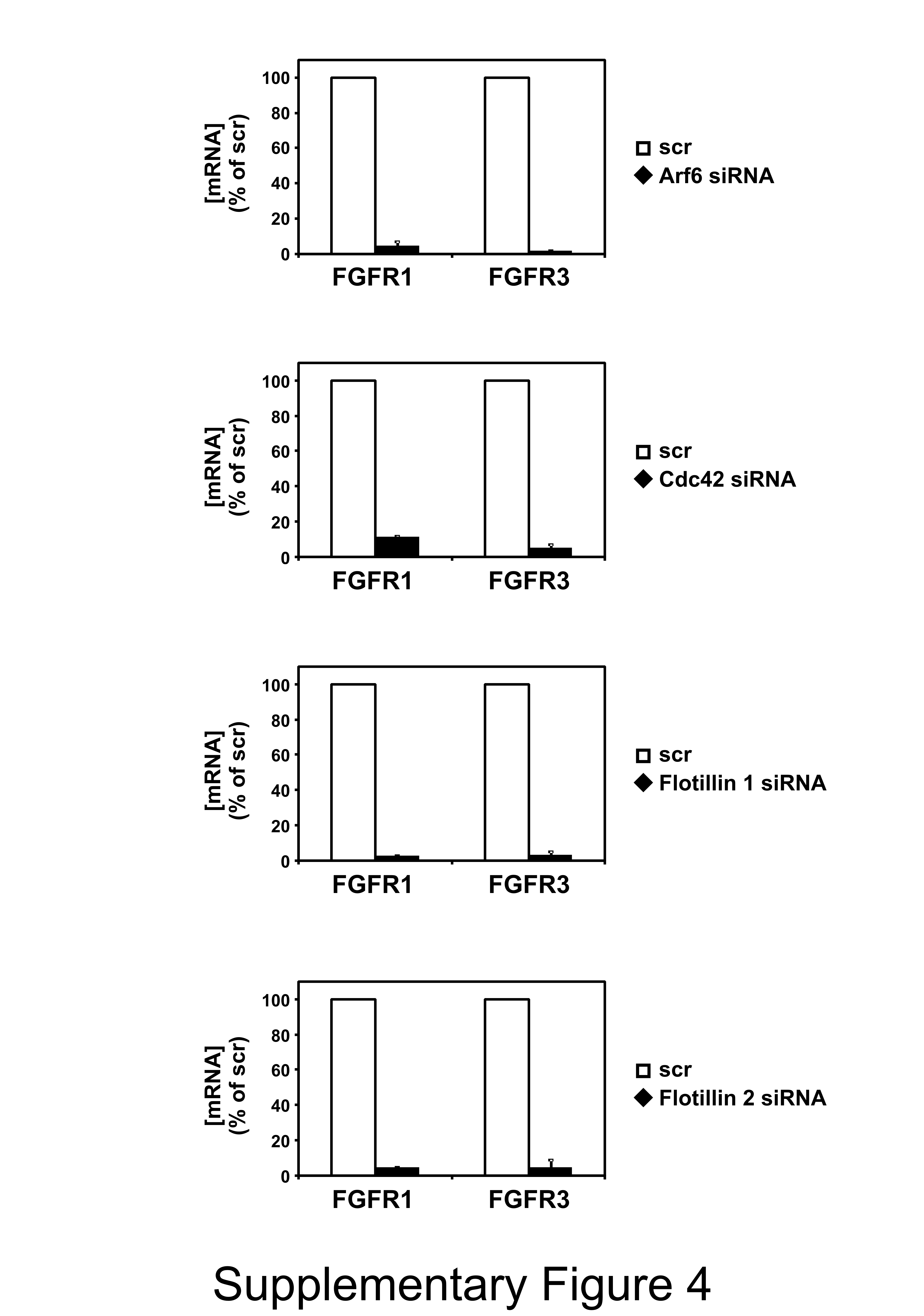

Supplement: Figure S4 — Knockdown efficiency examined by mRNA level. RNA isolation, cDNA synthesis and qRT-PCR were performed as described in materials and methods. The amount of mRNA were calculated relative to the housekeeping gene SDHA and are expressed as percentage of scr. The histograms represent the mean +s.d. of two independent experiments. (TIF) [file pone.0021708.s004.tif]

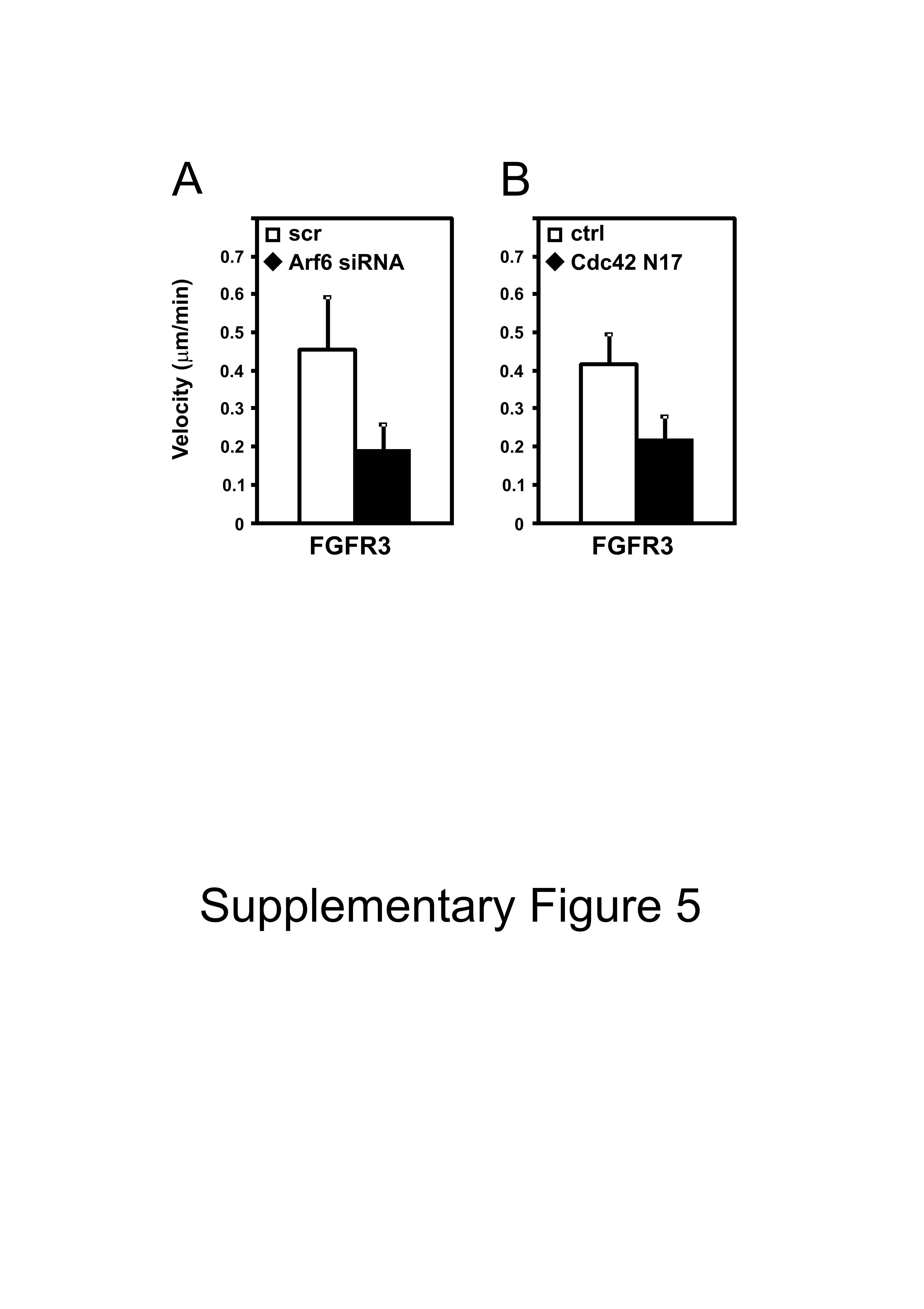

Supplement: Figure S5 — Validation of disruption of Arf6 and Cdc42. (A) U2OS cells stably expressing FGFR3 were transfected with siRNA oligos targeting Arf6 or a non-targeting siRNA control (scr) and monitored by imaging every 10 minutes for 9 hours in the presence of 100 ng/ml FGF1 and 20 U/ml heparin. The velocity of migration was quantified and the mean velocity is presented in the histogram. The histogram represents one independent experiment +s.d. and 39–40 cells were quantified for each condition. (B) U2OS cells stably expressing FGFR3 were transfected with EGFP-Cdc42 N17 and cell migration was monitored by imaging every 10 minutes for 15 hours in the presence of 100 ng/ml FGF1 and 20 U/ml heparin. The velocity of migration was quantified and the mean velocity is presented in the histogram. The histogram represents one independent experiment +s.d. and 10 cells were quantified for each condition. (TIF) [file pone.0021708.s005.tif]
